# Analysis of variation in NF-κB genes and expression levels of NF-κB-regulated molecules

**DOI:** 10.1186/1753-6561-1-s1-s126

**Published:** 2007-12-18

**Authors:** Wen Liu-Mares, Zhifu Sun, William R Bamlet, Elizabeth J Atkinson, Brooke L Fridley, Susan L Slager, Mariza de Andrade, Ellen L Goode

**Affiliations:** 1 Division of Epidemiology, Department of Health Sciences Research, Mayo Clinic College of Medicine, 200 First Street Southwest, Rochester, Minnesota 55905, USA; 2 Division of Biostatistics, Department of Health Sciences Research, Mayo Clinic College of Medicine, Rochester, 200 First Street Southwest, Harwick 7, Rochester, Minnesota 55905, USA

## Abstract

The nuclear factor-kappaB (NF-κB) family of transcription factors regulates the expression of a variety of genes involved in apoptosis and immune response. We examined relationships between genotypes at five NF-κB subunits (*NFKB1, NFKB2, REL, RELA*, and *RELB*) and variable expression levels of 15 NF-κB regulated proteins with heritability greater than 0.40: BCL2A1, BIRC2, CD40, CD44, CD80, CFLAR, CR2, FAS, ICAM1, IL15, IRF1, JUNB, MYC, SLC2A5, and VCAM1. SNP genotypes and expression phenotypes from pedigrees of Utah residents with ancestry from northern and western Europe were provided by Genetic Analysis Workshop 15 and supplemented with additional genotype data from the International HapMap Consortium. We conducted association, linkage, and family-based association analyses between each candidate gene and the 15 heritable expression phenotypes. We observed consistent results in association and linkage analyses of the *NFKB1 *region (encoding p50) and levels of FAS and IRF1 expression. FAS is a cell surface protein that also belongs to the TNF-receptor family; signals through FAS are able to induce apoptosis. IRF1 is a member of the interferon regulatory transcription factor family, which has been shown to regulate apoptosis and tumor-suppression. Analyses in the *REL *region (encoding c-Rel) revealed linkage and association with CD40 phenotype. CD40 proteins belong to the tumor necrosis factor (TNF)-receptor family, which mediates a broad variety of immune and inflammatory responses. We conclude that variation in the genes encoding p50 and c-Rel may play a role in NF-κB-related transcription of FAS, IRF1, and CD40.

## Methods

The nuclear factor-kappaB (NF-κB) family of transcription factors regulates the expression of hundreds of genes including pro-inflammatory and apoptosis genes [1-3]. Transcription of these genes is activated by five NF-κB subunits (*NFKB1 *encoding p50, *NFKB2 *encoding p52, *REL *encoding c-Rel, *RELA *encoding p65, and *RELB *encoding Rel-B). The NF-κB pathway is a critical candidate gene pathway for numerous cancers and cardiovascular endpoints.

### Samples and data availability

Genetic Analysis Workshop 15 (GAW15) Problem 1 included data on 14 three-generation pedigrees (two sets of grandparents, one set of parents, and a sibship of eight individuals) consisting of Utah residents with ancestry from northern and western Europe (CEPH-Utah, CEU). Pedigree members had genotypes on ~2882 single-nucleotide polymorphisms (SNPs) spread throughout the genome and ~3554 phenotypes consisting of expression levels from lymphoblastoid cells hybridized onto Affymetrix Genome Focus Arrays [4]. Expression density was scaled to 500 and transformed by log_2 _[4]. Forty-two participants (14 trios) were also studied by the International HapMap Consortium; thus, additional genotype data were available on selected individuals (including 28 unrelated individuals) in families 1340, 1341, 1345, 1346, 1347, 1362, 1408, 1416, and 1454 [5,6].

### Genotype selection

Genotypes from 21 GAW15-provided SNPs surrounding ~20 cM of each candidate gene were analyzed: *NFKB1 *(90.6 cM to 117.5 cM on chromosome 4), *NFKB2 *(94.5 cM to 119.8 cM on chromosome 10), *REL *(45.7 cM to 73.4 cM on chromosome 2), *RELA *(44.7 cM to 78.4 cM on chromosome 11), and *RELB *(41.2 cM to 58.0 cM on chromosome 19). Denser genotypes from HapMap within 5 kb of each gene were also used: *NFKB1 *(106 SNPs, mean *r*^2 ^= 0.25), *NFKB2 *(3 SNPs, mean *r*^2 ^= 0.01), *REL *(16 SNPs, mean *r*^2 ^= 0.41), *RELA *(3 SNPs, mean *r*^2 ^= 0.07), and *RELB *(8 SNPs, mean *r*^2 ^= 0.18).

### Phenotype selection and heritability

Regulatory targets of NF-κB transcription (*N *= 165) were compiled from review of the literature [1-3] and online catalogs [7]. Expression levels of 75 from these target genes were available in the GAW15 Problem 1 data. We estimated heritability (*h*^2^) using the Splus/R library *multic *[9] assuming a polygenic model in the 14 pedigrees. Fifteen phenotypes with *h*^2 ^greater than 0.4 (*p*-value < 0.001) were included in the current analysis (Table 1). Additional *h*^2 ^estimates are available upon request.

**Table 1:** Heritability (*h*^2^), association testing (minimum *p*-values of SNP and haplotype association test), and linkage analysis (maximum LOD scores)^a^

				*NFKB1 *(106 SNPs)			*NFKB2 *(3 SNPs)			*REL *(16 SNPs)			*RELA *(3 SNPs)		
							
Phenotype	Probe	*h*^2^	*h*^2 ^*p*-Value	SNP	Haplotype	LOD	SNP	Haplotype	LOD	SNP	Haplotype	LOD	SNP	Haplotype	LOD
BCL2A1	205681_at	0.42	0.0008	**0.029**	**0.040**	<1.00	**0.04**	>0.05	<1.00	>0.05	>0.05	**1.70**	>0.05	>0.05	**1.61**
BIRC2	202076_at	0.48	0.0003	>0.05	>0.05	<1.00	>0.05	>0.05	<1.00	>0.05	>0.05	<1.00	>0.05	>0.05	**1.24**
CD40	35150_at	0.49	0.0003	>0.05	>0.05	<1.00	**0.01**	>0.05	<1.00	**0.28**	**0.047**	**2.17**	>0.05	>0.05	**1.31**
CD44	204490_s_at	0.46	0.0004	**0.034**	**0.009**	<1.00	>0.05	>0.05	<1.00	>0.05	>0.05	<1.00	>0.05	>0.05	<1.00
CD80	207176_s_at	0.49	0.0003	**0.025**	**0.017**	<1.00	>0.05	>0.05	<1.00	>0.05	>0.05	<1.00	>0.05	>0.05	<1.00
CFLAR	211317_s_at	0.48	0.0003	>0.05	>0.05	<1.00	**0.05**	>0.05	<1.00	>0.05	>0.05	<1.00	>0.05	>0.05	<1.00
CR2	205544_s_at	0.47	0.0003	>0.05	>0.05	<1.00	**0.02**	>0.05	<1.00	>0.05	>0.05	<1.00	>0.05	>0.05	<1.00
FAS	204780_s_at	0.43	0.0007	**0.032**	>0.05	**1.38**	>0.05	>0.05	<1.00	>0.05	>0.05	<1.00	>0.05	>0.05	<1.00
ICAM1	202638_s_at	0.46	0.0004	**0.025**	**0.05**	<1.00	**0.05**	>0.05	<1.00	>0.05	>0.05	<1.00	>0.05	>0.05	<1.00
IL15	205992_s_at	0.41	0.0009	**0.013**	>0.05	<1.00	>0.05	>0.05	<1.00	**0.038**	**0.05**	<1.00	>0.05	>0.05	<1.00
IRF1	202531_at	0.42	0.0009	**0.031**	**0.022**	**2.54**	>0.05	>0.05	**1.45**	**0.01**	**0.05**	<1.00	>0.05	>0.05	<1.00
JUNB	201473_at	0.41	0.0009	**0.033**	>0.05	<1.00	**0.01**	>0.05	<1.00	**0.0082**	**0.016**	<1.00	>0.05	>0.05	<1.00
MYC	202431_s_at	0.51	0.0002	>0.05	>0.05	<1.00	>0.05	>0.05	<1.00	>0.05	>0.05	**1.37**	>0.05	>0.05	<1.00
SLC2A5	204429_s_at	0.47	0.0003	>0.05	>0.05	<1.00	**0.03**	>0.05	**1.01**	**0.02**	**0.002**	<1.00	>0.05	>0.05	<1.00
VCAM1	203886_s_at	0.43	0.0007	**0.011**	**0.010**	<1.00	>0.05	>0.05	<1.00	**0.01**	**0.048**	<1.00	>0.05	>0.05	<1.00

^a^No suggestive results were found for *RELB *genotypes (8 SNPs) and any phenotype. HapMap data was used for association testing; GAW15 data was used for linkage analysis.

^b^Bold indicates *p*-values of SNP or haplotype association test ≤0.05 or LOD score of linkage analysis >1.

### Linkage analysis in extended pedigrees

Variance components multipoint linkage analysis of 15 expression levels was performed using *multic *[9] with GAW15 genotype data among 14 extended pedigrees (194 individuals), assuming 1 Mb~1 cM.

### Family-based association

Family-based association tests (single-SNP and three-SNP haplotypes) were performed using the program *FBAT *[10] to examine the null hypothesis of no association and no linkage. Two analyses were conducted for each phenotype; first, dense HapMap genotypes in 14 trios, and second, GAW15 genotypes in 14 extended pedigrees.

### Association in unrelated individuals

Using data on 28 unrelated individuals, analysis of variance (ANOVA) tested associations between 15 heritable expression levels and genotypes at dense HapMap SNPs surrounding the five candidate genes. With the Splus library *HaploStat *[8], score testing assessed haplotype associations.

## Results

### Linkage analysis

Linkage analysis showed elevated LOD scores in the *NFKB1 *(FAS and IRF1 expression), *NFKB2 *(IRF1 and SLC2A5 expression), *REL *(CD40, BCL2A1, and MYC expression), and *RELA *regions (CD40, BCL2A1, and BIRC2 expression). Linkage regions and maximum LOD scores for each gene are presented in Tables 1 and 2.

**Table 2:** Linkage analysis and family-based association tests (FBAT) using GAW15 data

	Linkage analysis		FBAT (*p *< 0.05)	
		
Gene	Max LOD (cM)	cM LOD > 1	SNPs^a^	Haplotypes^a^
*NFKB1 *(chromosome 4)				
FAS	1.38 (101.0)	97.4–106.8	rs721412	rs765220-rs971696-rs721412
IRF1	2.54 (106.7)	94.0–109.0		rs971696-rs721412-rs1557803
				
*NFKB2 *(chromosome 10)				
IRF1	1.45 (102.1)	100.0–112.0	---	---
SLC2A5	1.01 (97.5)	97.5–98.2		
				
*REL *(chromosome 2)				
CD40	2.17 (70.2)	62.4–73.7	rs1363062	rs1520446-rs1974771-rs1363062
BCL2A1	1.70 (70.2)	67.0–73.7	rs1106577	rs1177274-rs2167564-rs1106577
MYC	1.37 (47.7)	45.0–50.0		rs2167564-rs1106577-rs2216924
				
*RELA *(chromosome 4)				
CD40	1.31 (60.6)	60.6–62.4	rs1867791	rs1966864-rs1993205-rs1867791
BCL2A1	1.61 (60.8)	60.6–65.1		rs1867791-rs999297-rs175110
BIRC2	1.24 (61.0)	60.6–63.8		
				
*RELB *(chromosome 19)				
FAS	0.66 (58.0)	---	---	---

^a^For each gene, SNPs and haplotypes were the same for all the phenotypes shown.

### Family-based association tests (FBAT)

Analyses using GAW15-provided genotypes surrounding *NFKB1 *suggested an association between rs721412 at 111.3 cM and FAS, IRF1 expression. Haplotypes containing this SNP were also associated with FAS and IRF1 expression (Table 2). Using the HapMap data we found rs4648134 at 103.9 cM associated with CD80, FAS and ICAM1 phenotypes across three different methods (association, linkage, and FBAT) (Table 3). Analysis of *REL *GAW15 data revealed associations between genotypes at rs1363062 and rs1106577 and CD40, BCL2A1, and MYC expression levels (Table 2). FBAT analysis of denser *REL *HapMap data did not suggest any association with SNPs or haplotypes and any phenotype (Table 4). Using GAW15 data in the *RELA *region, we found that genotypes of rs1867791 at 44.9 cM had FBAT *p*-values of 0.02. Haplotype FBAT analysis indicated that two haplotypes were point-wise significantly associated with CD40, BCL2A1, and BIRC2 expression (Table 2). Using HapMap data, genotypes at rs11820062 were associated with each phenotype (*p*-values~0.02), and haplotype rs2306365-rs732072-rs11820062 was associated with all phenotypes (*p*-values~0.03).

**Table 3:** Association analysis of *NFKB1 *using HapMap data^a^

Phenotype	SNP	*p*-Value	Haplotype	*p*-Value
BCL2A1	rs17032779	0.035	rs17032779-rs230519-rs93059	0.040
				
CD44	rs230506	0.034	rs230506-rs230505-rs230504	0.009
	rs3774934	0.034	rs3774933-rs3774934-rs4647972	0.037
				
CD80	rs4648091	0.025	rs4648090-rs4648091-rs4648095	0.049
	rs4648134	0.025	rs4648133-rs4648134-rs4648135	0.017
				
FAS	rs4648134	0.032	---	---
				
ICAM1	rs4648134	0.032	rs4648133-rs4648134-rs4648135	0.050
				
IRF1	rs1598859	0.032	rs1610152-rs1598859-rs3774956	0.040
	rs3774959	0.032	rs3821958-rs1020759-rs3774959	0.040
				
VCAM1	rs7679591	0.011	rs230528-rs7679591-rs230526	0.040
	rs17032779	0.014	rs230521-rs230520-rs17032779	0.047
	rs4648018	0.014	rs4648016-rs4648018-rs230500	0.046
	rs4648069	0.014	rs4648055-rs4648068-rs4608069	0.049
	rs4648091	0.014	rs4648090-rs4648091-rs4648095	0.010
	rs4648134	0.014	rs4648133-rs4648134-rs4648135	0.050
	rs10489114	0.014	rs3774959-rs10489114-rs7377680	0.034
	rs4648015	0.014	rs230496-rs4648015-rs230498	0.041
	rs4648016	0.014	rs230498-rs4648016-rs4648018	0.046
	rs4648043	0.014	rs3774956-rs4648043-rs3821958	0.037

^a^Family-based association tests suggested SNP rs4648136 and haplotype rs4648134-rs4648135-rs4648136 associated with CD80, FAS and ICAM1 phenotypes.

**Table 4:** Association analysis of *REL *using HapMap data^a^

Phenotype	SNP	*p*-Value	Haplotype	*p*-Value
CD40	rs13422089	0.028	rs6545835-rs10208155-rs13422089	0.070
				
IL15	rs842648	0.038	rs13422089-rs842648-rs13022703	0.050
				
IRF1	rs842644	0.010	rs842644-rs6545836-rs10193964	0.050
				
JUNB	rs6545835	0.047	rs6545835-rs10208155-rs13422089	0.050
	rs10208155	0.047	---	---
	rs13422089	0.008	rs13422089-rs842648-rs13022703	0.048
	rs10185028	0.047	rs10185028-rs842647-rs842644	0.027
				
SLC2A5	rs6545835	0.019	rs6545835-rs10208155-rs13422089	0.020
	rs10208155	0.019	rs6545835-rs10208155-rs13422089	0.020
	rs13422089	0.035	rs6545835-rs10208155-rs13422089	0.020
	rs10185028	0.019	rs10185028-rs842647-rs842644	0.004
	rs842644	0.047	rs10185028-rs842647-rs842644	0.004
	rs6545836	0.019	rs842647-rs842644-rs6545836	0.004
	rs10193964	0.019	rs842644-rs6545836-rs10193964	0.002
				
VCAM1	rs842644	0.010	rs842644-rs6545836-rs10193964	0.048

^a^Family-based association tests did not suggest associations with the above phenotypes.

### Association in unrelated individuals

We examined associations between 15 expression phenotypes and genotypes at HapMap SNPs. Haplotype analyses indicated an overlap with the single SNP association results for *NFKB1 *and *REL *(Table 1). Among nine phenotypes associated with SNPs in *NFKB1*, six (BCL2A1, CD44, CD80, ICAM1, IRF1, and VCAM1) had suggestive haplotype associations. Among six phenotypes associated with *REL *SNPs, all six phenotypes had suggestive haplotype association (Table 1). More detailed results are available upon request.

## Discussion

We utilized a variety of methods (association, linkage, and family-based association) in an attempt to understand the relationship between variation in NF-κB genes and expression levels of 15 proteins. We consider this to be an exploratory analysis of publicly available data with a limited sample size. We sought to reveal avenues for future study within the NF-κB pathway. As an assessment of these methods, we concluded that haplotype analysis combined with single-SNP analysis, family-based association tests, and linkage analysis has helped inform our understanding of the NF-κB pathway. Analyses revealed association and linkage between *NFKB1 *and FAS, IRF1 expression phenotypes, and between *REL *and CD40 expression phenotype. FAS is a cell surface protein that belongs to the tumor necrosis factor (TNF) receptor family; signals through FAS are able to induce apoptosis. IRF1 is a member of the interferon regulatory transcription factor family, which regulates apoptosis and tumor-suppression. CD40 proteins also belong to TNF protein family, which is essential in mediating a broad variety of immune and inflammatory responses. Based upon our results, we concluded that variation in the *NFKB1 *and *REL *genes may play a role in downstream regulation of FAS, IRF1, and CD40 expression.

There are several limitations to this study, including lack of adjustment for multiple tests on multiple loci and use of a small sample size; interpretation of tests on a sample of 14 warrants caution. No results were statistically significant after taking into account the multiple comparisons. Nonetheless, these exploratory analyses provide clues for further large scale studies.

## Conclusion

We make three general conclusions. First, single-SNP association testing was less conservative than haplotype and FBAT analysis, where haplotype analyses indicated association, results of single-SNP association testing were also significant; however, association found by single-SNP testing was not always revealed by haplotype analysis. Because this is not simulated data, we do not know whether the single-SNP results represent true or false positives. Second, because haplotype analysis requires two or more SNPs, for those genes with only one or very few SNPs, haplotype analysis might not be an appropriate analysis to perform. Third, FBAT analysis was relatively conservative compared to single-SNP and haplotype association analyses. FBAT found fewer SNPs and haplotypes with point-wise significance. In summary, we suggest that single-SNP and haplotype association analyses be used in first-stage analysis to generate a smaller set of candidate SNPs; FBAT and linkage analysis can then narrow down the list of potentially important loci.

## Competing interests

The author(s) declare that they have no competing interests.
